# Long noncoding RNA landscapes specific to benign and malignant thyroid neoplasms of distinct histological subtypes

**DOI:** 10.1038/s41598-021-96149-2

**Published:** 2021-08-18

**Authors:** Valentina D. Yakushina, Vladimir V. Strelnikov, Alexander S. Tanas, Alexander V. Lavrov

**Affiliations:** Research Centre for Medical Genetics, Moscow, Russia

**Keywords:** Cancer genomics, Endocrine cancer, Tumour biomarkers

## Abstract

The main types of thyroid neoplasms, follicular adenoma (FA), follicular thyroid carcinoma (FTC), classical and follicular variants of papillary carcinoma (clPTC and fvPTC), and anaplastic thyroid carcinoma (ATC), differ in prognosis, progression rate and metastatic behaviour. Specific patterns of lncRNAs involved in the development of clinical and morphological features can be presumed. LncRNA landscapes within distinct benign and malignant histological variants of thyroid neoplasms were not investigated. The aim of the study was to discover long noncoding RNA landscapes common and specific to major benign and malignant histological subtypes of thyroid neoplasms. LncRNA expression in FA, FTC, fvPTC, clPTC and ATC was analysed with comprehensive microarray and RNA-Seq datasets. Putative biological functions were evaluated via enrichment analysis of coexpressed coding genes. In the results, lncRNAs common and specific to FTC, clPTC, fvPTC, and ATC were identified. The discovered lncRNAs are putatively involved in L1CAM interactions, namely, pre-mRNA processing (lncRNAs specific to FTC); PCP/CE and WNT pathways (lncRNAs specific to fvPTC); extracellular matrix organization (lncRNAs specific to clPTC); and the cell cycle (lncRNAs specific to ATC). Known oncogenic and suppressor lncRNAs (RMST, CRNDE, SLC26A4-AS1, NR2F1-AS1, and LINC00511) were aberrantly expressed in thyroid carcinomas. These findings enhance the understanding of lncRNAs in the development of subtype-specific features in thyroid cancer.

## Introduction

The major types of thyroid cancer are papillary carcinoma (PTC) and follicular carcinoma (FTC), accounting for approximately 70–80% and 10–15% of all thyroid cancers, respectively. Both PTC and FTC are derived from follicular cells and are well-differentiated thyroid cancers (WDTCs), with distinct mutational landscapes, clinical behaviours, typical sites of metastasis and prognostic clinical markers^1^. Within PTC, several variants can be distinguished, and classical (clPTC) and follicular variants (fvPTC) are the most frequently identified. fvPTC has intermediate behaviour; it is composed of neoplastic follicles rather than papillae, but with follicular cells showing nuclear features characteristic of PTC. The benign counterpart of FTC is follicular adenoma (FA), and it is often challenging to differentiate them through cytology. FA, FTC and fvPTC compose follicular-pattern thyroid tumours, sharing common mutational prevalence and clinical features^1,2^. Anaplastic thyroid carcinoma (ATC) is the most advanced and aggressive thyroid cancer and the least likely to respond to treatment^3,4^. Based on the differences in mutational landscapes, morphology and clinical behaviour of histological subtypes, specific molecular patterns, including patterns of long noncoding RNAs (lncRNAs), are expected to be associated with these features.

Evidence of the important roles of lncRNAs in tumour suppression, cancer progression, invasion and metastatic potential and their prognostic and therapeutic value is increasing^5^. LncRNAs are RNA molecules of more than 200 nucleotides that typically do not have a functional open reading frame (however, bifunctional RNAs have been discovered that function as both protein-coding and noncoding RNAs). Many lncRNA genes have two or more exons and display 5′-capping, polyadenylation and alternative splicing. The functions of lncRNAs are realized in different ways: recruiting transcription factors, chromatin organizers, or chromatin modifiers, forming DNA–RNA triplex anchoring effector proteins to the gene promoter, acting as decoys for miRNAs and proteins, or interfering with protein posttranslational modification^5–8^. Relative to the coding genes, lncRNAs can be classified into intergenic (lincRNA); antisense (on the opposite strand of a protein-coding locus); sense intronic or overlapping (on the same strand, with transcript in introns of a coding gene, or containing a coding gene in its intron); retained intron (an alternatively spliced transcript containing an intronic sequence); bidirectional (originates from the promoter region of a protein-coding gene with transcription proceeding in the opposite direction on the other strand); and 3-prime overlapping (overlap the 3′UTR of a protein-coding locus on the same strand). Today, the number of annotated lncRNA genes has reached 14 720 according to Ensembl version 93^9^.

In thyroid cancer, several lncRNAs have been shown to have pathogenic and predictive roles, including BANCR, FALEC, CNALPTC1, PVT1, NAMA, PTCSC1, PTCSC2, PTCSC3, and TNRC6C-AS1^10–21^. However, all of the studies to date have considered only PTC, and mostly none of the previous works takes into account the difference between clPTC and fvPTC. There are no published studies describing the landscapes of lncRNAs in ATC, FTC and FA. Nevertheless, lncRNAs differentially expressed in ATC may reflect anaplastic features and be strong prognostic factors. As the morphology and behaviour of FTC differ from those of PTC, it is proposed that the landscape of lncRNAs in FTC may be different from that of PTC. Investigation of lncRNAs common and specific to FA and FTC is important in understanding their relations and revealing differential diagnostic markers.

This study aimed to identify lncRNAs specific and common to the main types of thyroid neoplasms (FA, FTC, fvPTC, clPTC and ATC). The expression data from microarray technology (8 datasets) and RNA-Seq technology (the PRJEB11591 dataset and TCGA transcriptome data) were analysed.

## Results

### LncRNAs differentially expressed in thyroid neoplasms

LncRNA expression was evaluated in the main histological subtypes of thyroid neoplasms, FA, FTC, fvPTC, clPTC, and ATC, compared to those in NT. The expression of 3910 lncRNA genes in the microarray dataset, 2587 in the RNA-Seq PRJEB11591 dataset and 3009 in the RNA-Seq TCGA dataset was analysed. The number of genes analysed corresponded to the total number of lncRNAs covered by uniquely mapped probes in the Affymetrix Human Genome U133 Plus 2.0 Array and the number of lncRNAs yielded after filtration by low number of counts for RNA-Seq datasets.

The numbers of lncRNAs found to be differentially expressed in each subtype compared to NT are presented in Table 1. The complete lists of the identified differentially expressed lncRNAs are shown in Supplementary files 1–3. Volcano plots representing the distribution of fold change and adjusted p values in the studied histological subtypes are shown in Supplementary file 4.

**Table 1:** Numbers of lncRNAs differentially expressed in thyroid nodules compared to normal thyroid tissue.

Histological type of the nodule	Microarray	RNA-Seq PRJEB11591	RNA-Seq TCGA
FA		143	
FTC		213	
fvPTC	84	213	174
clPTC	137	401	308
ATC	330		

*FA* follicular adenoma, *FTC* follicular thyroid carcinoma, *fvPTC* follicular variant of papillary thyroid carcinoma, *clPTC* classical variant of papillary thyroid carcinoma, *ATC *anaplastic carcinoma.

**Figure 1:**
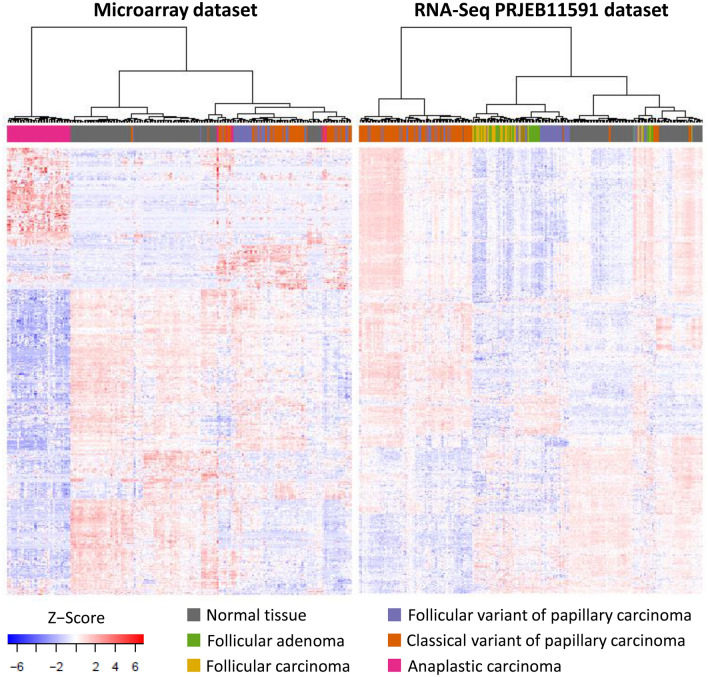
Clustering of FA, FTC, fvPTC, clPTC and ATC by the expression of lncRNA. (**A**) Microarray dataset; (**B**) RNA-Seq PRJEB11591 dataset. Genes differentially expressed in each histological subtype are included.

Hierarchical clustering of differentially expressed lncRNAs in the microarray and PRJEB11591 datasets is presented in Fig. 1. There was strong clustering of ATC, clustering of clPTC and weak clustering of fvPTC lncRNAs. No clustering of lncRNAs within the FTC or FA groups was observed (Fig. 1).

**Figure 2:**
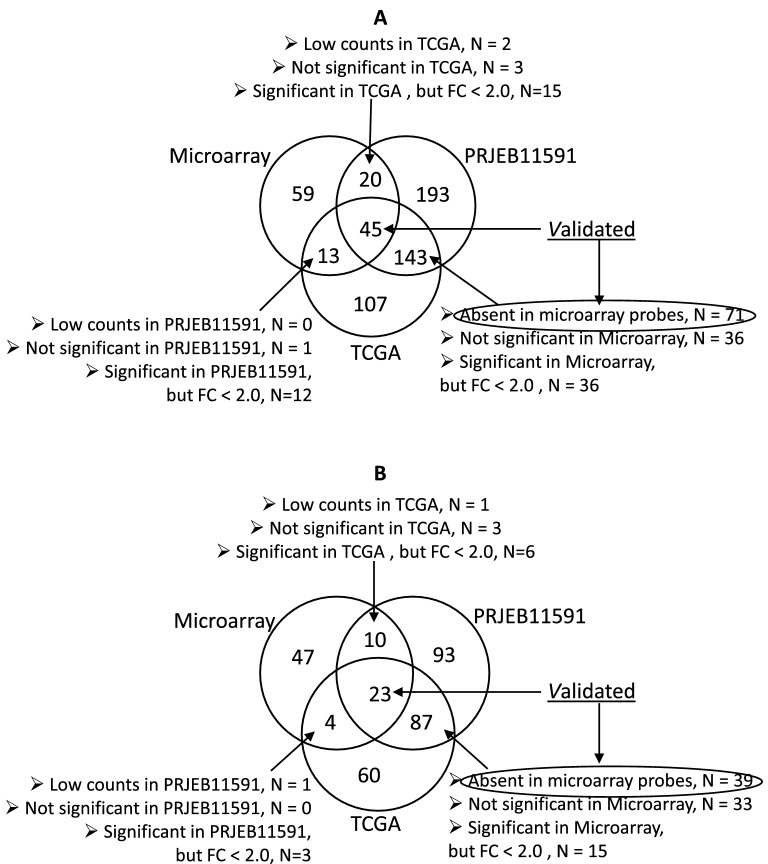
In silico validation of differentially expressed lncRNAs in clPTC (**A**) and fvPTC (**B**). In clPTC, 116 genes were considered to be validated (differentially expressed in all datasets or differentially expressed in both RNA-Seq datasets but absent in microarray probes). In fvPTC, 62 genes were considered to be validated.

Via cross-dataset confirmation, 116 genes in clPTC were validated (45 genes found in all analysed datasets, 71 genes without probes in the microarray were found in both RNA-Seq datasets; Fig. 2A), and 62 genes in fvPTC were validated (Fig. 2B). These genes can be considered to have robustly differentially expressed lncRNAs. There are no datasets available for performing cross-dataset validation of FA, FTC or ATC genes.

**Figure 3:**
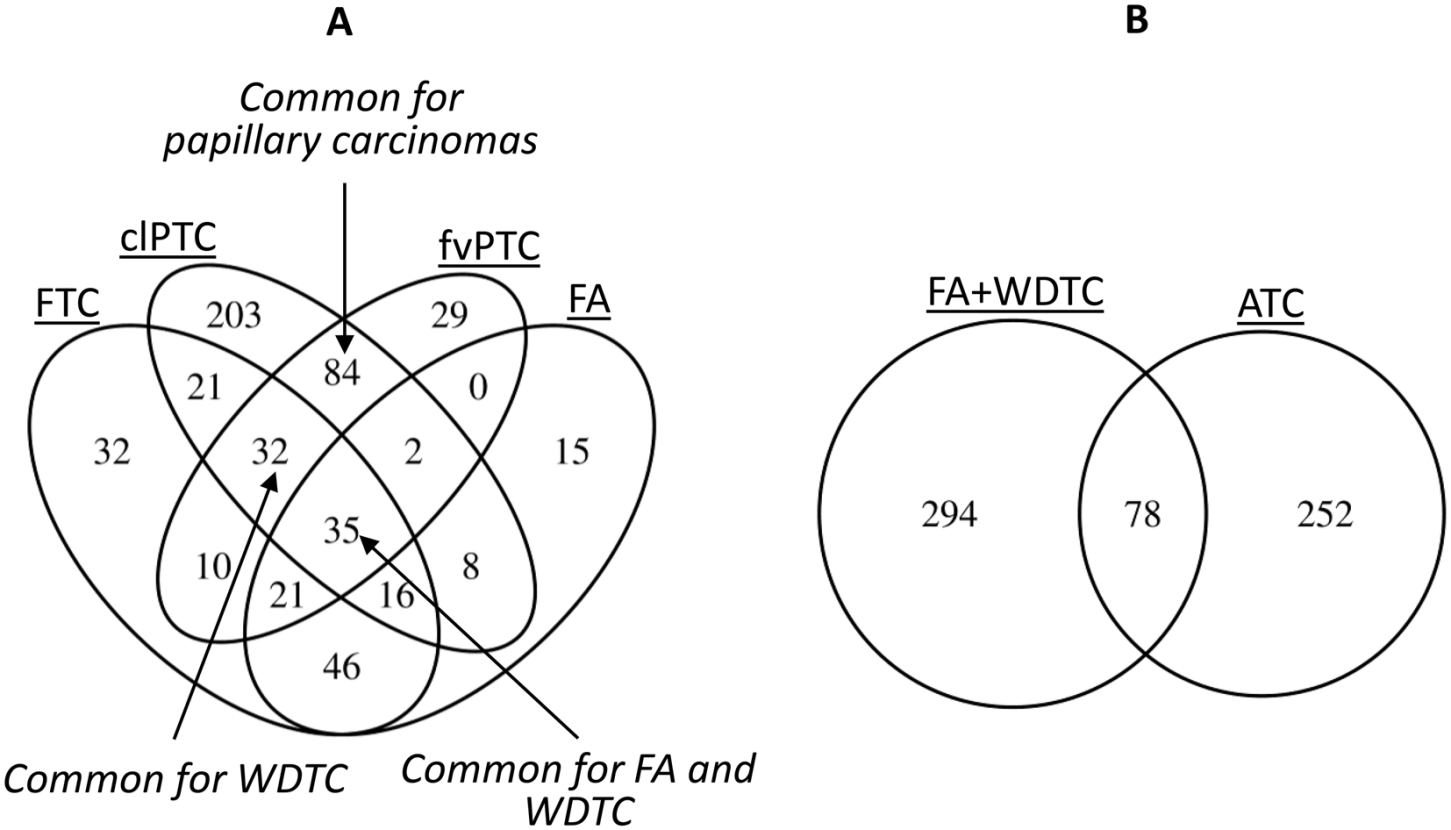
Intersection of lncRNA landscapes in thyroid neoplasms. (**A**) lncRNA landscapes in FA, FTC, fvPTC, and clPTC (based on the RNA-Seq PRJEB11591 dataset). (**B**) LncRNA landscapes in differentiated neoplasms (total list of lncRNAs differentially expressed in FA, FTC, fvPTC, and clPTC) and ATC.

**Figure 4:**
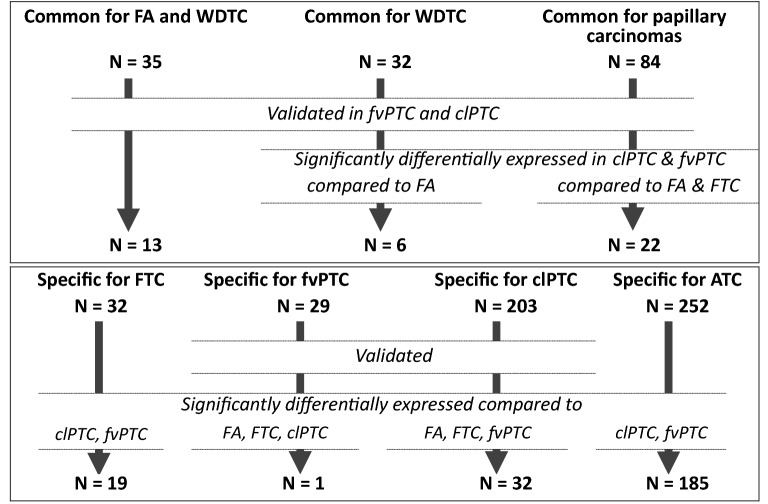
Numbers of common and specific lncRNAs found after intersection analysis and filtration.

LncRNAs common and specific to each histological subtype were detected via intersection of the genes expressed differentially in each subtype compared to NT, and subsequent selection of lncRNAs validated in clPTC and fvPTC, and significantly differentially expressed in comparison between subtypes of neoplasms (Figs. 3, 4).

### LncRNAs common to FA and WDTC

Of the 35 lncRNAs found to be differentially expressed in FA and WDTC (FTC, clPTC, and fvPTC) compared to NT, 13 genes were cross validated in clPTC and fvPTC (Figs. 3, 4, Table 2). The expression of LINC02555 and LINC02471 increased during the progression from adenoma to carcinomas and was significantly higher in fvPTC and clPTC than in FA. The expression of ENSG00000256542 and ENSG00000258117 decreased during the transition from FA to carcinomas and was significantly lower in fvPTC and clPTC than in FA or FTC.

**Table 2:** LncRNAs common for FA and WDTC.

ENSG ID	HGNC symbol	Gene biotype	Log(FC) compared to NT			
			FA	FTC	fvPTC	clPTC
ENSG00000223914	LINC02471	lincRNA	2	4.2	7.2^ab^	6.5^ab^
ENSG00000260943	LINC02555	lincRNA	2.7	4.8	7.8^ab^	5^ac^
ENSG00000234546	LINC01759	lincRNA	1.5	1.5	1.8	1.5
ENSG00000237813	–	antisense	− 2	− 1.7	− 2.6	− 1.3^c^
ENSG00000259104	PTCSC3	lincRNA	− 2	− 2	− 1.6	− 1.3
ENSG00000248810	LINC02432	lincRNA	− 1.2	− 1.2	− 2.2	− 1.5
ENSG00000167912	–	antisense	− 1.8	− 2.3	− 2.6	− 1.5^c^
ENSG00000259884	–	lincRNA	− 2.2	− 2.6	− 2.5	− 1.6
ENSG00000226816	–	lincRNA	− 1.9	− 3.3	− 2.3	− 1.6^b^
ENSG00000237863	–	antisense	− 1.7	− 1.1	− 1.5	− 2.5^bc^
ENSG00000206129	–	lincRNA	− 2.2	− 1.9	− 2.8	− 3^b^
ENSG00000256542	–	antisense	− 1.4	− 1.9	− 3.3^ab^	− 3.2^ab^
ENSG00000258117	–	lincRNA	− 1.9	− 2.6	− 4.3^ab^	− 4.3^ab^

Differential expression of all genes is validated in clPTC and fvPTC. ^a^Significantly differentially expressed compared to FA; ^b^significantly differentially expressed compared to FTC; ^c^significantly differentially expressed compared to fvPTC.

### LncRNAs common to WDTC

There were 32 lncRNAs differentially expressed in all the studied histological subtypes of WDTC (FTC, clPTC, and fvPTC) but not in FA (Fig. 3). Of these lncRNAs, 6 lncRNAs were validated to be significantly differentially expressed in clPTC and fvPTC compared to FA (Fig. 4, Table 3). None of the 32 lncRNAs were differentially expressed in FTC compared to FA.

**Table 3:** LncRNAs common for WDTC.

ENSG ID	HGNC symbol	Gene biotype	Log(FC) compared to NT		
			FTC	fvPTC	clPTC
ENSG00000256268	LINC02454	lincRNA	2.0	4.2^a^	4.5^a^
ENSG00000225342	–	antisense	1.7	4.4^a^	3.1^ab^
ENSG00000250343	STK32A-AS1	antisense	1.6	3.1^a^	3.2^a^
ENSG00000272384	–	lincRNA	1.1	1.9	2.5^a^
ENSG00000233251	–	antisense	− 1.4	− 2.3	− 1.6
ENSG00000254489	–	antisense	− 1.8	− 3.2^a^	− 3.9^a^

Differential expression of all genes is validated in clPTC and fvPTC. Expression in fvPTC and clPTC differs significantly compared to FA. ^a^significantly differentially expressed compared to FTC; ^b^significantly differentially expressed compared to fvPTC.

### LncRNAs common to papillary carcinomas

There were 22 genes differentially expressed in both clPTC and fvPTC, but not in FA or FTC (Fig. 3), validated and significantly differentially expressed compared to FA and FTC (Fig. 4, Table 4)—lnRNAs are putatively associated with papillary features in thyroid carcinomas.

**Table 4:** LncRNAs common for papillary carcinomas.

ENSG ID	HGNC symbol	Gene biotype	Log(FC) compared to NT	
			fvPTC	clPTC
ENSG00000237463		Antisense	4.2	6.4
ENSG00000203585	LINC02408	lincRNA	2.0	4.8
ENSG00000251002		Antisense	4.2	4.7
ENSG00000272482		lincRNA	1.7	3.6
ENSG00000204282	TNRC6C-AS1	Antisense	2.4	3.4
ENSG00000197301		Antisense	2.4	3.4
ENSG00000267199		Antisense	1.7	3.1
ENSG00000235978		Antisense	2.2	2.5
ENSG00000230910		Antisense	2.0	2.2
ENSG00000257989		lincRNA	2.5	2.2
ENSG00000224020	MIR181A2HG	Antisense	1.7	1.8
ENSG00000272079		lincRNA	2.1	1.7
ENSG00000272512		lincRNA	1.0	1.6
ENSG00000237742		Antisense	1.9	1.4
ENSG00000255366		lincRNA	1.1	1.3
ENSG00000265666	RARA-AS1	Antisense	1.4	1.0
ENSG00000204934	ATP6V0E2-AS1	Antisense	− 1.1	− 1.5
ENSG00000228559		lincRNA	− 1.2	− 1.6
ENSG00000234899	SOX9-AS1	lincRNA	− 1.2	− 1.7
ENSG00000228613		Antisense	− 2.2	− 2.6
ENSG00000267034		lincRNA	− 2.6	− 2.9
ENSG00000261399		Antisense	− 2.0	− 3.3

These lncRNAs are validated and differentially expressed compared to FA and FTC.

### LncRNA specific to histological subtypes of differentiated carcinomas

Nineteen lncRNAs were aberrantly expressed in FTC but not in the other studied neoplasms and were significantly differentially expressed compared to those in clPTC and fvPTC (Figs. 3, 4, Table 5). However, none of these lncRNAs was differentially expressed compared to those in FA.

**Table 5:** LncRNAs specific for FTC.

ENSG ID	HGNC symbol	Gene biotype	Log(FC) compared to NT
ENSG00000281383	^–^	lincRNA	1.4
ENSG00000272732	^–^	lincRNA	− 1.0
ENSG00000224660	SH3BP5-AS1	Antisense	− 1.0
ENSG00000225855	RUSC1-AS1	Antisense	− 1.0
ENSG00000197989	SNHG12	Antisense	− 1.0
ENSG00000198221	AFDN-DT	lincRNA	− 1.1
ENSG00000248019	FAM13A-AS1	Antisense	− 1.1
ENSG00000273576	^–^	lincRNA	− 1.1
ENSG00000261087	^–^	lincRNA	− 1.1
ENSG00000271895	^–^	Antisense	− 1.2
ENSG00000242282	^–^	lincRNA	− 1.2
ENSG00000272374	^–^	lincRNA	− 1.2
ENSG00000204584	^–^	Antisense	− 1.3
ENSG00000262370	^–^	lincRNA	− 1.3
ENSG00000205959	^–^	lincRNA	− 1.3
ENSG00000285103	^–^	Bidirectional_promoter_lncRNA	− 1.4
ENSG00000276007	^–^	Sense_intronic	− 1.4
ENSG00000226419	SLC16A1-AS1	Antisense	− 1.5
ENSG00000257671	KRT7-AS	Antisense	− 1.6

None of these lncRNAs is significantly differentially expressed compared to FA.

Of the 29 genes differentially expressed in fvPTC but not in other differentiated carcinomas or FA (Figs. 3, 4), only the ENSG00000257647 gene was specific to fvPTC-validated and significantly differentially expressed in fvPTC compared to FA, FTC and clPTC.

The 32 genes were found to be differentially expressed in clPTC but not in other differentiated carcinomas or FA, validated, and significantly differentially expressed compared to those in fvPTC, FTC and FA-lncRNAs specific to clPTC (Figs. 3, 4, Table 6).

**Table 6:** LncRNAs specific for clPTC.

ENSG ID	HGNC symbol	Gene biotype	Log(FC) compared to NT
ENSG00000227036	LINC00511	lincRNA	2.5
ENSG00000237187	NR2F1-AS1	Antisense	2.5
ENSG00000260604	–	lincRNA	2.2
ENSG00000262903	–	Antisense	2.1
ENSG00000261101	–	Sense_overlapping	1.9
ENSG00000274021	–	Antisense	1.8
ENSG00000281406	BLACAT1	lincRNA	1.5
ENSG00000245571	FAM111A-DT	lincRNA	1.3
ENSG00000253930	TNFRSF10A-AS1	Antisense	1.3
ENSG00000235609	–	lincRNA	1.2
ENSG00000237943	PRKCQ-AS1	lincRNA	− 1.0
ENSG00000260572	–	Antisense	− 1.0
ENSG00000204860	FAM201A	Antisense	− 1.0
ENSG00000177640	CASC2	Antisense	− 1.1
ENSG00000259704	–	Sense_overlapping	− 1.1
ENSG00000231769	–	Antisense	− 1.2
ENSG00000231231	LINC01423	lincRNA	− 1.2
ENSG00000272622	–	lincRNA	− 1.2
ENSG00000251602	–	Antisense	− 1.3
ENSG00000231856	–	Antisense	− 1.3
ENSG00000249249	–	Antisense	− 1.3
ENSG00000205791	LOH12CR2	lincRNA	− 1.3
ENSG00000232415	ELN-AS1	Antisense	− 1.5
ENSG00000262185	–	Sense_overlapping	− 1.8
ENSG00000224885	EIPR1-IT1	Sense_intronic	− 1.9
ENSG00000256151	ADGRD1-AS1	lincRNA	− 1.9
ENSG00000231107	LINC01508	lincRNA	− 2.0
ENSG00000267128	RNF157-AS1	Antisense	− 2.0
ENSG00000229457	LINC01789	lincRNA	− 2.0
ENSG00000249487	LINC01586	lincRNA	− 2.7
ENSG00000224568	LINC01886	lincRNA	− 2.7
ENSG00000233705	SLC26A4-AS1	Antisense	− 3.0

### LncRNA specific to ATC

ATC samples were available only in the microarray dataset, which also included two variants of PTC. Of the 376 lncRNAs differentially expressed in ATC compared to NT, 252 were not differentially expressed in the other investigated histological subtypes, and 185 were significantly differentially expressed compared to those in clPTC and fvPTC-lncRNAs specific to ATC. The 30 most differentially expressed genes are presented in Table 7, and the full list is shown in Supplementary file 5.

**Table 7:** Top 30 lncRNAs specific for ATC.

ENSG ID	HGNC symbol	Gene biotype	Log(FC) compared to NT
ENSG00000272872	–	Sense_intronic	3.5
ENSG00000244158	–	Antisense	3.2
ENSG00000245694	CRNDE	lincRNA	3.2
ENSG00000240476	LINC00973	lincRNA	3.1
ENSG00000282638	–	lincRNA	3.0
ENSG00000247134	–	lincRNA	3.0
ENSG00000254615	–	lincRNA	2.9
ENSG00000280018	–	lincRNA	2.8
ENSG00000233682	–	antisense	− 2.5
ENSG00000266904	LINC00663	lincRNA	− 2.6
ENSG00000228506	–	Antisense	− 2.6
ENSG00000275234	–	Antisense	− 2.7
ENSG00000232229	LINC00865	lincRNA	− 2.8
ENSG00000269609	RPARP-AS1	lincRNA	− 2.9
ENSG00000270820	–	Antisense	− 2.9
ENSG00000271474	–	Antisense	− 3.0
ENSG00000284644	–	Antisense	− 3.0
ENSG00000273015	–	lincRNA	− 3.1
ENSG00000260686	–	Sense_overlapping	− 3.2
ENSG00000180769	WDFY3-AS2	Antisense	− 3.2
ENSG00000247400	DNAJC3-DT	lincRNA	− 3.2
ENSG00000271858	–	Antisense	− 3.3
ENSG00000236155	–	Processed_transcript	− 3.3
ENSG00000224078	SNHG14	Antisense	− 3.5
ENSG00000250073	–	Antisense	− 3.8
ENSG00000203709	MIR29B2CHG	lincRNA	− 4.1
ENSG00000261183	SPINT1-AS1	Antisense	− 4.2
ENSG00000229891	LINC01315	lincRNA	− 4.3
ENSG00000257151	PWAR6	lincRNA	− 4.8
ENSG00000255794	RMST	lincRNA	− 5.7

### Potential biological functions of aberrantly expressed lncRNAs

The coexpressed genes for each lncRNA from the top 5 most differentially expressed list for discussed groups were identified. The number of coexpressed genes was 138.5 (46.25 − 256.5) −median (Q1 − Q3).

**Figure 5:**
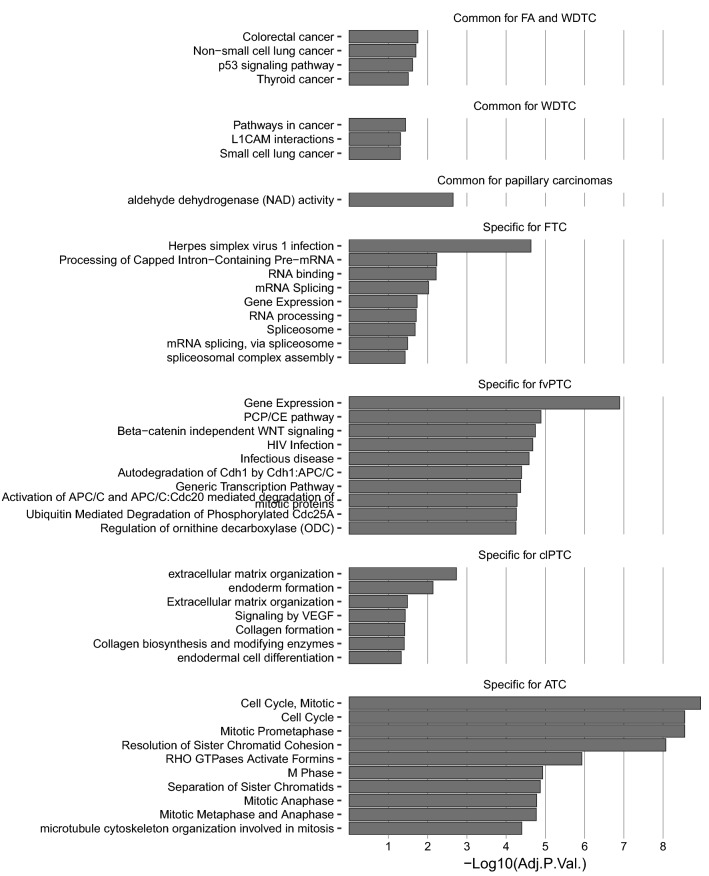
Putative biological process involving aberrantly expressed lncRNAs in thyroid neoplasms. The enrichment analysis of GO Biological Process, GO Molecular Function, KEGG, and Reactome terms was performed for the 5 most differentially expressed lncRNAs, and terms with adjusted p values ≤ 0.05 were considered significantly enriched. For fvPTC and ATC, the 10 most significantly enriched terms are presented.

Analysis of the enrichment of GO biological processes and GO molecular functions, KEGG, and Reactome terms with coexpressed coding genes allowed us to identify putative pathways involving common and specific lncRNAs (Fig. 5). The main functions of the lncRNAs common to FA and WDTC-cancers (Colorectal, Non-small cell lung, Thyroid) and p53 signalling; functions of the lncRNAs common to WDTC-Pathways in cancer and L1CAM interactions; functions of the lncRNAs common to papillary carcinomas-aldehyde dehydrogenase (NAD) activity; functions of the lncRNAs specific to FTC-processing of capped intron-containing pre-mRNA; functions of the lncRNAs specific to fvPTC-PCP/CE pathway and Beta-catenin independent WNT signalling; functions of the lncRNAs specific to clPTC-extracellular matrix organization and endoderm formation; and functions of the lncRNAs specific to ATC-cell cycle and mitotic processes.

## Discussion

Histological subtypes of follicular cell-derived thyroid carcinomas (FTC, PTC, and ATC) significantly differ in their mutational landscapes and clinical characteristics. Although FTC and clPTC are both WDTCs, FTC is characterized by a follicular growth pattern and tends more often to spread as metastases to distant organs, while clPTC typically has papillary architecture and spreads more often to lymph nodes in the neck. In FTC, K/H/NRAS and PAX8/PPARG mutations are prevalent, whereas BRAF mutations and tyrosine kinase fusions prevail in clPTC^1^. The clinical characteristics of fvPTC are intermediate; fvPTC is composed of neoplastic follicles not papillae, but with follicular cells showing nuclear features typical of PTC^22^. The mutational profile of fvPTC is most similar to that of FTC: both exhibit a prevalence of K/H/NRAS and PAX8/PPARG mutations. In a previous TCGA study, fvPTC was characterized as a Ras-like tumour, and its classification as a papillary carcinoma was questioned^23^. Recently, reclassification of encapsulated fvPTC as a “noninvasive follicular thyroid neoplasm with papillary-like nuclear features” (NIFTP) was proposed^2^. ATC is an advanced stage thyroid neoplasm and is the most aggressive thyroid cancer. It is expected that there are specific molecular features, including lncRNA patterns, associated with the clinical and histological features of WDTC and the aggressive behaviour of ATC. FA is thought to be a benign counterpart of FTC, and understanding the common and different molecular features of these neoplasms is important for the development of diagnostic and therapeutic strategies.

In this study, the expression of lncRNAs was evaluated in the main histological subtypes of thyroid neoplasms: FA, FTC, fvPTC, clPTC and ATC. Datasets analysed in the study (a microarray dataset of 8 independent experiments; RNA-Seq PRJEB11591; and RNA-Seq TCGA) allowed us to perform robust cross-dataset validation of the results for clPTC and fvPTC and to include representative sets of FA, FTC and ATC samples. LncRNA landscapes in FA, FTC and ATC were analysed for the first time. The highest number of genes aberrantly expressed compared to normal thyroid tissue were found in ATC (330 lncRNAs), followed by clPTC, FTC and fvPTC, which reflects the more advanced stage of ATC. Since the data for ATC, FA and FTC wer limited with one dataset only, the results for these subtypes are preliminary.

Intersection of the differentially expressed lncRNAs and subsequent comparison of the expression between subtypes of neoplasms led to the discovery of lncRNAs common to FA and WDTC (13 genes), common to WDTC (6 genes), common to classical and follicular variants of PTC (22 genes), and specific to FTC (19 genes), fvPTC (1 gene), clPTC (32 genes), and ATC (185 genes). The discovered lncRNAs were proposed to be involved in the development of clinical and morphological features of the studied subtypes. Putative biological processes involving common and specific lncRNAs were identified.

LncRNAs common to all studied thyroid neoplasms (including FA) and common to WDTC appear to be involved in carcinogenesis in different locations. L1CAM interactions found in this study to involve lncRNAs common to WDTC have been previously associated with well-described roles in tumour progression, metastases and the epithelial-to-mesenchymal transition^24,25^.

LncRNAs common to follicular and classical variants of papillary carcinoma (associated with papillary histology) are involved in aldehyde dehydrogenase (NAD) activity. Aldehyde dehydrogenase is known to maintain cancer stem cell properties in various cancers, including the thyroid^26^.

Biological processes involving lncRNAs specific to FTC include processes that are associated with splicing (Processing of Capped Intron-Containing Pre-mRNA, mRNA Splicing, and RNA processing). Accumulating evidence suggests that aberrant RNA splicing is a common and driving event in cancer development and progression. For instance, oncogenic Ras signalling via the ERK and PI3-K/Akt pathways regulates the phosphorylation of splicing factors such as SRSF1, SRSF7, and SPF45 and drives the switching of active and inactive states of tumour promoters and suppressors (MST1R, FAS, CD44, LBR, Casp-9, KLF6, and others) via alternative splicing^27,28^. None of the lncRNAs specific to FTC were differentially expressed compared to those in FA. The absence of lncRNAs differentially expressed in FTC and FA corresponds to the commonality of these subtypes and frequent difficulty in cytology-based differential diagnostics.

Only lncRNA ENSG00000257647 is specific to fvPTC, which might be explained by its intermediate morphology with features of both papillary and follicular carcinomas, leading to its debatable classification. LncRNA ENSG00000257647 appeared to be involved in WNT signalling, predominantly through the Beta-catenin-independent WNT pathway (especially, planar cell polarity that modulates cytoskeleton rearrangements through the activation of the small GTPases RhoA and Rac and their downstream effectors Rock and JNK). WNT signalling is known to play a crucial role in thyroid carcinogenesis, and several mechanisms of its deregulation have been described, including inhibition of the β-catenin degradation complex via its phosphorylation by RET/PTC, inhibition of E-cadherin expression through the MAPK/ERK pathway activated by BRAF mutations, and activation of both canonical and non-canonical Wnt pathways by RAS mutations^29,30^.

LncRNAs specific to clPTC are involved in extracellular matrix organization and endoderm and collagen formation. Extracellular matrix (ECM) disorganization is known to play a pivotal role in cancer initiation and progression. The major driving mutation in clPTC is BRAF p.V600E, and there is emerging evidence of ECM remodelling induced by BRAF p.V600E in PTCs^31^. Notably, it has been previously shown that the extracellular matrix of PTCs driven by BRAF p. V600E (but not mutant HRAS) is enriched with stromal-derived fibrillar collagen and facilitates cancer progression^32^.

lncRNAs specific to ATC are probably associated with its anaplastic features and aggressive behaviour. For these lncRNAs, there is a strong enrichment of cell cycle and mitotic pathways which possibly reflects the involvement of these lncRNAs in the loss of differentiation and high proliferation rate characteristic of ATC.

Of lncRNAs previously described in thyroid cancer, we found that PTCSC3 was downregulated in all investigated neoplasms, including FA; TNRC6C-AS1 was upregulated in papillary carcinomas; and PVT1 was specifically upregulated in ATC^11,17,18^. Other lncRNAs previously described in thyroid malignancy (BANCR, NAMA, CNALPTC1, FALEC, and PTCSC2) were not identified in our study^10,12,13,16,20,21^. A possible explanation is the strong association of these lncRNAs with specific mutations and the heterogeneity of driving mutations within the same subtype; for example, the aberrant expression of BANCR is driven by BRAF mutation.

The aberrant expression of lncRNAs with Ensembl annotation found by Liyanarachchi et al. (2016) in PTC was confirmed in our study^14^. Most of these lncRNAs were common to thyroid neoplasms (including FA) or common to classical and follicular variants of PTC. No lncRNAs found in our study to be subtype specific were discovered by Liyanarachchi.

In the present study, we identified some lncRNAs with known roles in tumorigenesis but not previously described in thyroid cancer^33-37^. The identified upregulated promoters of cancer progression included NR2F1-AS1 and LINC00511 in clPTC and CRNDE in ATC; downregulated tumour suppressors SLC26A4-AS1 in clPTC and RMST in ATC.

## Conclusion

LncRNAs common to FA and WDTC, common to WDTC, common to carcinomas with papillary features, and specific to clPTC, fvPTC, FTC and ATC were discovered in the analysis performed with the most comprehensive datasets (combination of a microarray dataset and two RNA-Seq datasets). The similarity of the lncRNA landscapes in FTC and FA was revealed. The results showed that LncRNAs common to FA and WDTC and common to WDTC are involved in pathways in cancer at various sites, p53 signalling and L1CAM interactions; lncRNAs common to papillary carcinomas are involved in aldehyde dehydrogenase (NAD) activity; lncRNAs specific to FTC are involved in mRNA processing; a lncRNA specific to fvPTC is involved in planar cell polarity and WNT signalling; lncRNAs specific to clPTC are involved in extracellular matrix organization and endoderm formation; and lncRNAs specific to ATC are involved in the cell cycle and mitotic processes; and LncRNAs found to be specific to ATC, including CRNDE and RMST, are likely associated with cancer aggressiveness and cancer progression.

## Materials and methods

### Microarray datasets

The microarray datasets obtained from Affymetrix Human Genome U133 Plus 2.0 Array (Platform GPL570) were originally selected from the GEO database. The following datasets were included: GSE3467, GSE60542, GSE35570, GSE76039, GSE53157, GSE33630, GSE65144, and GSE29265. A total of 107 samples of normal tissue (NT) and 32 fvPTC, 48 clPTC, and 49 ATC samples were analysed. CEL files were downloaded, and normalization was performed using the gcrma R package. Microarray probes were annotated with Ensembl version 93 using the biomaRt package^38^.

### RNA-Seq datasets

*The* RNA-Seq dataset PRJEB11591 of Yoo et al.^39^ was selected from the EBI European Nucleotide Archive database (https://www.ebi.ac.uk/ena/data/view/PRJEB11591). PRJEB11591 is the most comprehensive available RNA-Seq dataset containing benign and malignant thyroid neoplasms (FA, FTC, fvPTC, and clPTC). The PRJEB11591 samples included 81 NT, 26 FA, 30 FTC, 48 fvPTC and 77 clPTC samples. FASTQ files were downloaded, and alignment was performed by HISAT2^40^. Counts were calculated using featureCounts (Rsubread package) with annotation by Ensembl version 93 and Ensemble gene ID for grouping attributes^41^. Genes with low counts (less than 2 counts in number of samples exceeding the size of the smallest sample group) were eliminated, and TMM normalization (edgeR package) and the voom method using the limma R package were applied.

In the TCGA transcriptome data, 58 NT, 356 clPTC and 101 fvPTC were selected. Samples of metastases and other minor histological subtypes were excluded. Raw counts (HTSeq-Counts Workflow Type, briefly, STAR 2-pass alignment followed by gene expression count assessment with HTSeq) were downloaded from Genomic Data Commons Data Portal (GDC, https://portal.gdc.cancer.gov/). Genes with low counts (less than 1 count in number of samples exceeding the size of smallest sample group) were eliminated, followed by TMM normalization (edgeR package) and voom analysis with limma^42^.

### Selection of lncRNA genes

Protein-coding genes and genes attributed to Havana biotypes not related to lncRNAs were eliminated. Genes of the following Havana biotypes were included in the analysis: lincRNA, antisense, 3-prime overlapping ncRNA, bidirectional promoter lncRNA, misc RNA, processed transcript, sense intronic, and sense overlapping.

### Statistical analysis

To identify differentially expressed lncRNAs, linear modelling using the limma package was performed^43^. Genes with FDR adjusted p value ≤ 0.01 and fold change (FC) ≥ 2.0 were considered to be differentially expressed. A heat map analysis of differentially expressed genes was performed using coolmap limma.

### Validation

For clPTC and fvPTC, the sets of genes found to be significantly differentially expressed in a previous step in the microarray, RNA-Seq PRJEB11591, and RNA-Seq TCGA datasets were processed with intersection. Genes found in all three datasets and genes found in both RNA-Seq datasets but not in microarray probes were considered validated.

### Selection of lncRNAs common and specific to histological subtypes

LncRNAs common and specific to FA and WDTC were selected via the intersection analysis of genes found to be significantly differentially expressed in each subtype compared to NT in the RNA-Seq dataset PRJEB11591 and subsequent application following criteria:

- Common to FA and WDTC-confirmed through validation for clPTC and fvPTC;
- Common to WDTC-confirmed through validation for clPTC and fvPTC, and significantly differentially expressed compared to FA;
- Common to papillary carcinomas-confirmed through validation for clPTC and fvPTC, and significantly differentially expressed compared to FA and FTC;
- Specific to clPTC, fvPTC, FTC-confirmed through validation for clPTC and fvPTC (not applied to FTC), and significantly differentially expressed compared to each studied subtype;

LncRNAs specific to ATC were selected from intersection with genes found in FA and WDTC with subsequent filtration of genes significantly differentially expressed compared to those in clPTC and fvPTC.

### Evaluation of potential biological functions

To identify genes positively and negatively coexpressed with 5 most differentially expressed lncRNAs, pairwise Pearson correlation between the lncRNAs and all the genes was calculated using the RNA-Seq PRJEB11591 dataset (for FA, FTC, fvPTC and clPTC) and the microarray dataset (for ATC). Genes with an absolute r ≥ 0.7 and a significant correlation (p value < 0.05) were considered to be coexpressed. For coexpressed genes, enrichment of Gene Ontology (GO) Biological Process (2018), GO Molecular Function (2018), Kyoto Encyclopedia of Genes and Genomes (KEGG, 2019) and Reactome (2016) terms was estimated using Enrichr^44,45^. Terms with adjusted p values in Fisher's exact test ≤ 0.05 were considered significantly enriched^42–46^.

## Supplementary Information

Supplementary Information 1.

Supplementary Information 2.

Supplementary Information 3.

Supplementary Information 4.

Supplementary Information 5.
