# Supplementary material for: Long noncoding RNA landscapes specific to benign and malignant thyroid neoplasms of distinct histological subtypes
Source: Sci Rep. 2021 Aug 18;11:16728. doi: 10.1038/s41598-021-96149-2 (PMC8373968; doi:10.1038/s41598-021-96149-2)
Supplement: Supplementary file 4 — Supplementary Information 4. [file 41598_2021_96149_MOESM4_ESM.pdf]

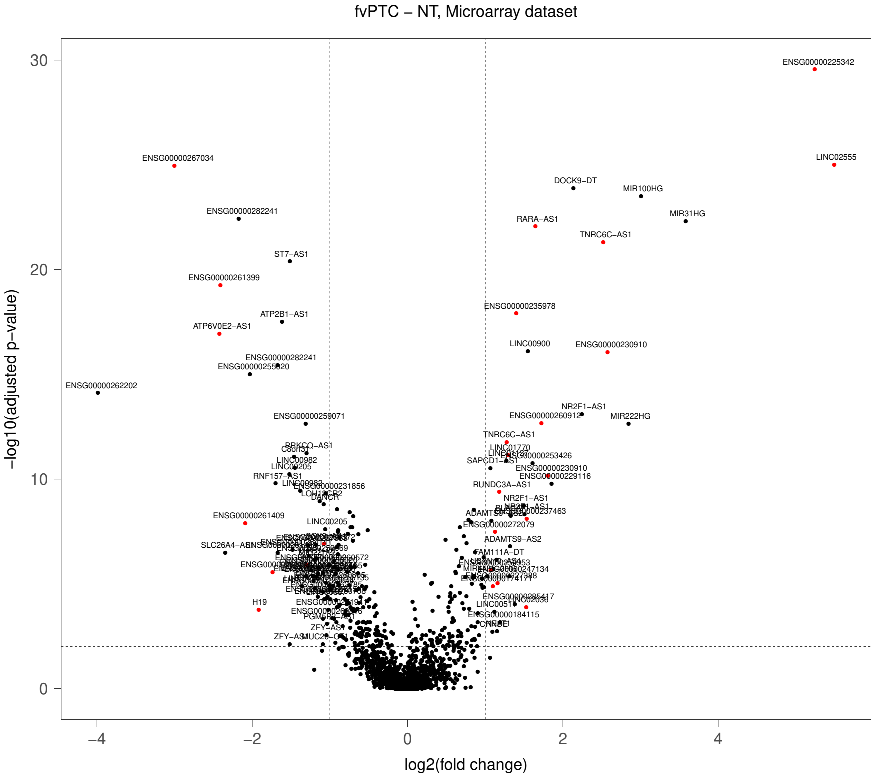

Red highlights lncRNA differently expressed in Microarray, RNASeq PRJEB11591 and TCGA datasets

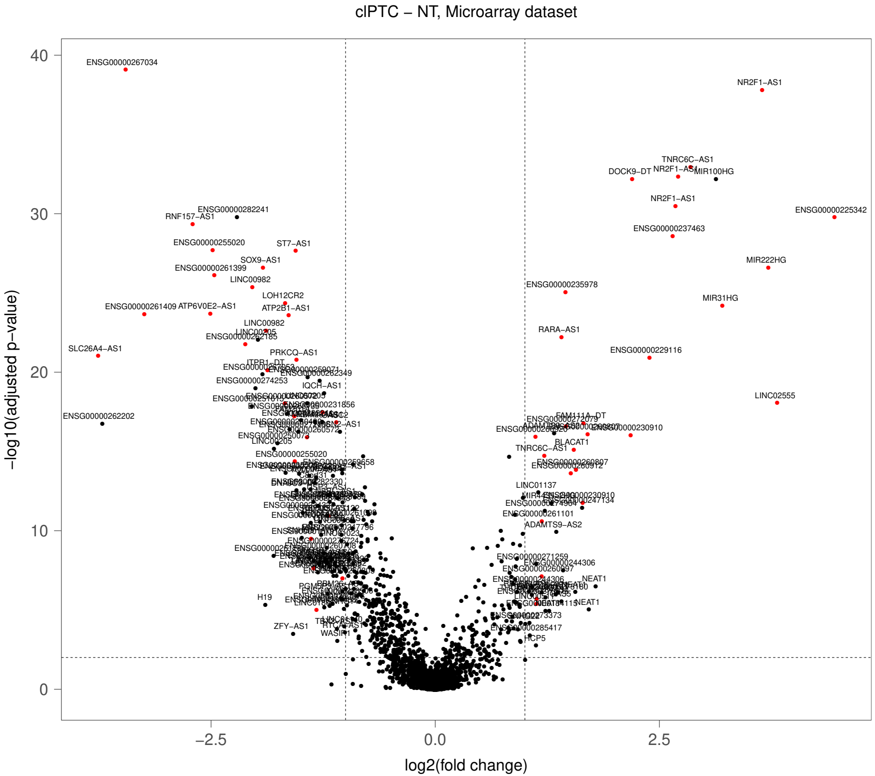

Red highlights lncRNA differently expressed in Microarray, RNASeq PRJEB11591 and TCGA datasets

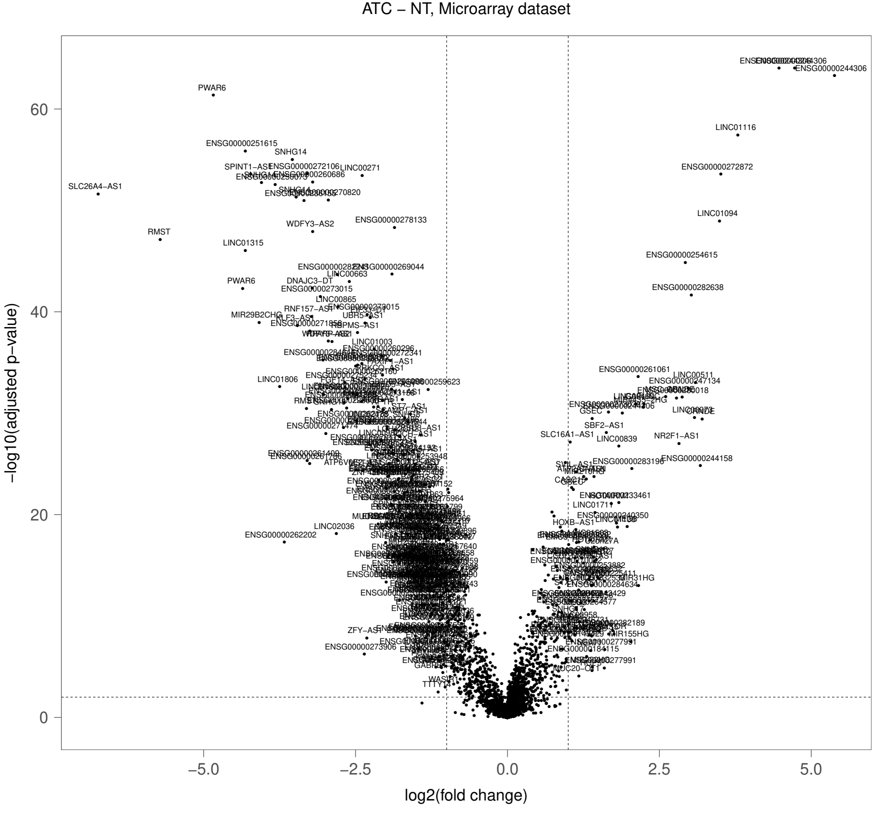
ATC − NT, Microarray dataset
−log10(adjusted p−value)
log2(fold change)
0
20
40
60
−5.0
−2.5
0.0
2.5
5.0
PWAR6
SLC26A4−AS1
RMST
LINC01315
ENSG00000251615
SNHG14
SPINT1−AS1
LINC00271
ENSG00000272106
ENSG00000260686
WDFY3−AS2
ENSG00000278133
ENSG00000269044
LINC00663
DNAJC3−DT
ENSG00000273015
LINC00865
RNF157−AS1
UBR5−AS1
MIR29B2CHG
RBPMS−AS1
LINC01003
LINC01806
ENSG00000261409
LINC02036
ENSG00000262202
ZFY−AS1
ENSG00000273906
WASIR1
TTTY14
ENSG00000244306
LINC01116
ENSG00000272872
LINC01094
ENSG00000254615
ENSG00000282638
ENSG00000261061
LINC00511
ENSG00000247134
LINC00973
CRNDE
NR2F1−AS1
SBF2−AS1
SLC16A1−AS1
LINC00839
GSEC
ENSG00000283196
ENSG00000244158
LINC01711
ENSG00000240350
HOXB−AS1
SNHG17
ENSG00000282189
MIR155HG
ENSG00000277991
ENSG00000184115
MUC20−OT1

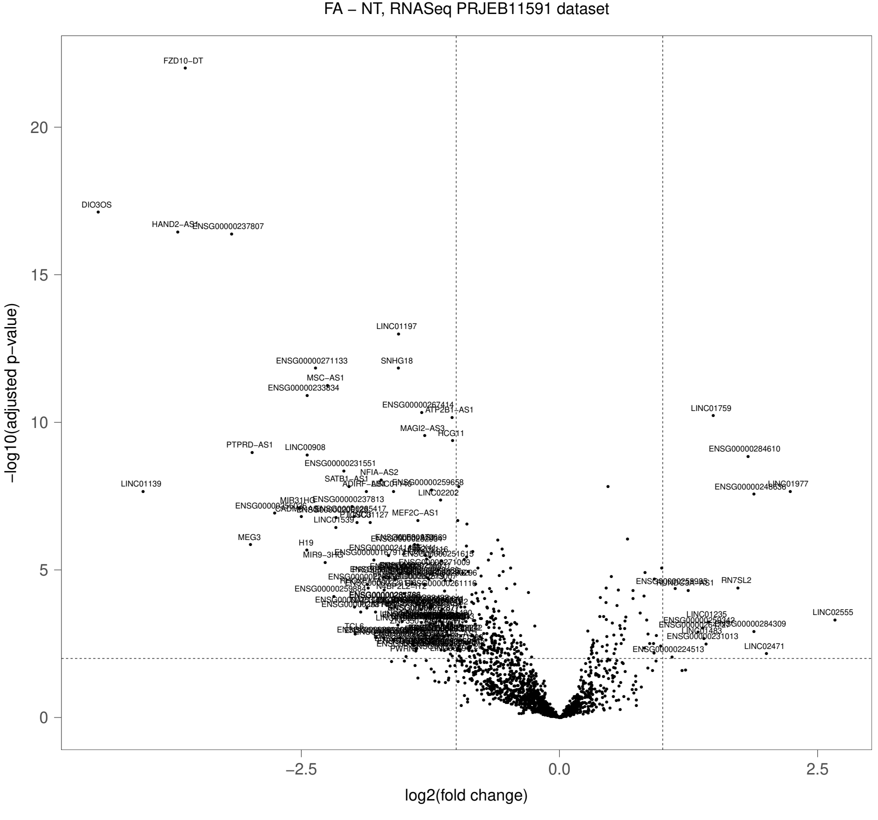

FA − NT, RNASeq PRJEB11591 dataset
−log10(adjusted p−value)
log2(fold change)
0
5
10
15
20
−2.5
0.0
2.5
FZD10−DT
DIO3OS
HAND2−AS1
ENSG00000237807
LINC01197
ENSG00000271133
SNHG18
MSC−AS1
ENSG00000267414
ATP2B1−AS1
MAGI2−AS3
HCG11
PTPRD−AS1
LINC00908
ENSG00000231551
NFIA−AS2
SATB1−AS1
ENSG00000259658
LINC01139
LINC02202
MIR31HG
ENSG00000237813
LINC01539
MEF2C−AS1
MEG3
H19
MIR9−3HG
ENSG00000271009
ENSG00000261116
ENSG00000259884
TCL6
PWRN1
LINC01759
ENSG00000284610
LINC01977
RN7SL2
LINC02555
LINC01235
ENSG00000284309
LINC01483
ENSG00000231013
ENSG00000224513
LINC02471

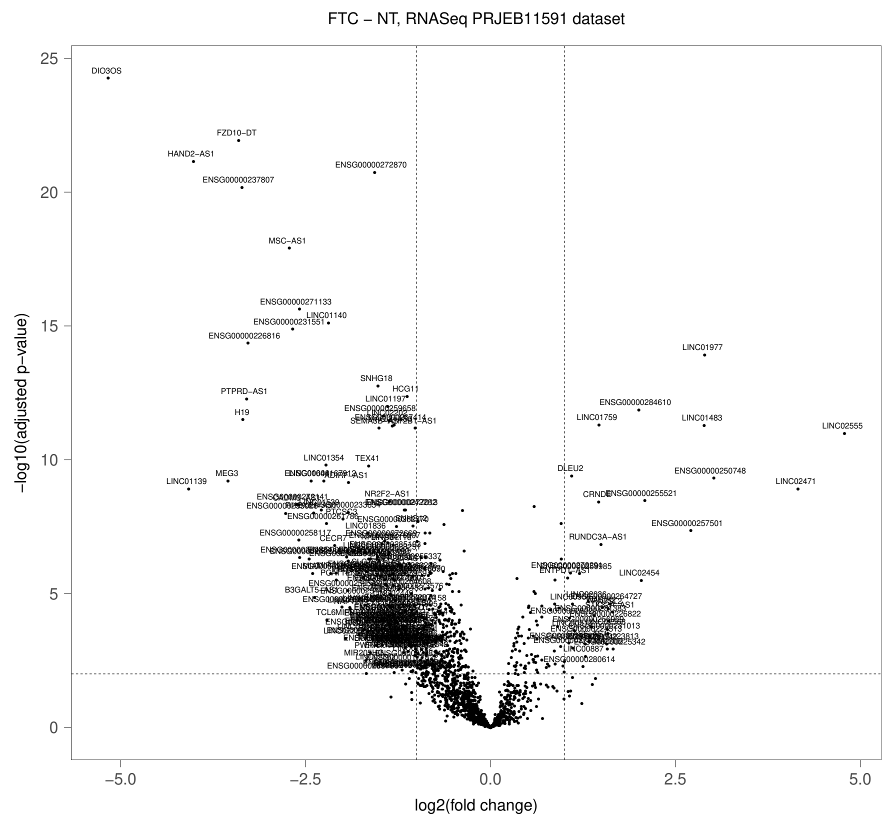
FTC – NT, RNASeq PRJEB11591 dataset
–log10(adjusted p–value)
log2(fold change)
25
20
15
10
5
0
–5.0
–2.5
0.0
2.5
5.0
DIO3OS
FZD10–DT
HAND2–AS1
ENSG00000272870
ENSG00000237807
MSC–AS1
ENSG00000271133
LINC01140
ENSG00000231551
ENSG00000226816
LINC01977
SNHG18
HCG11
PTPRD–AS1
LINC01197
ENSG00000284610
H19
LINC01759
LINC01483
LINC02555
LINC01354
TEX41
DLEU2
ENSG00000250748
MEG3
LINC01139
LINC02471
NR2F2–AS1
CRNDE
ENSG00000255521
ENSG00000261786
PTCSC3
ENSG00000258117
LINC01836
ENSG00000257501
RUNDC3A–AS1
CECR7
ENTPD1–AS1
LINC02454
B3GALT5–AS1
LINC00887
ENSG00000280614

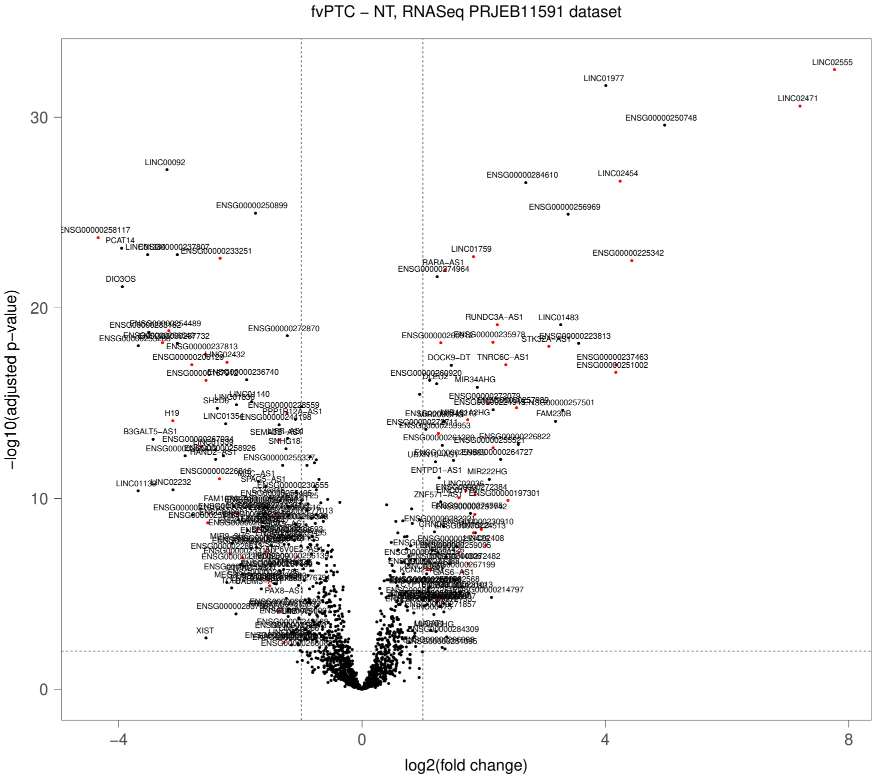

Red highlights lncRNA differently expressed in Microarray, RNASeq PRJEB11591 and TCGA datasets

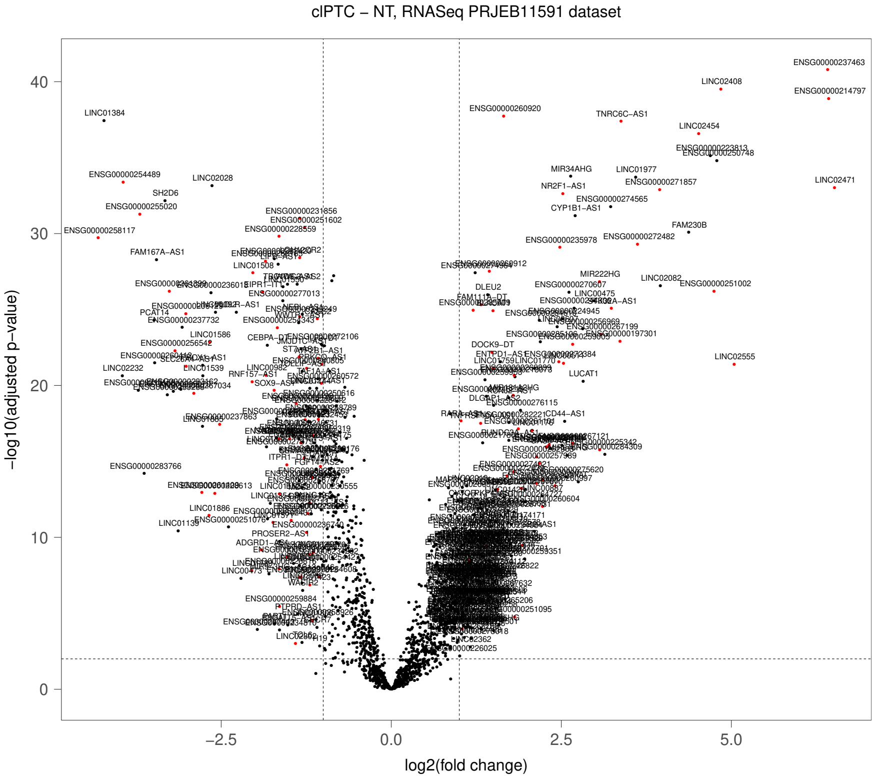

Red highlights lncRNA differently expressed in Microarray, RNASeq PRJEB11591 and TCGA datasets

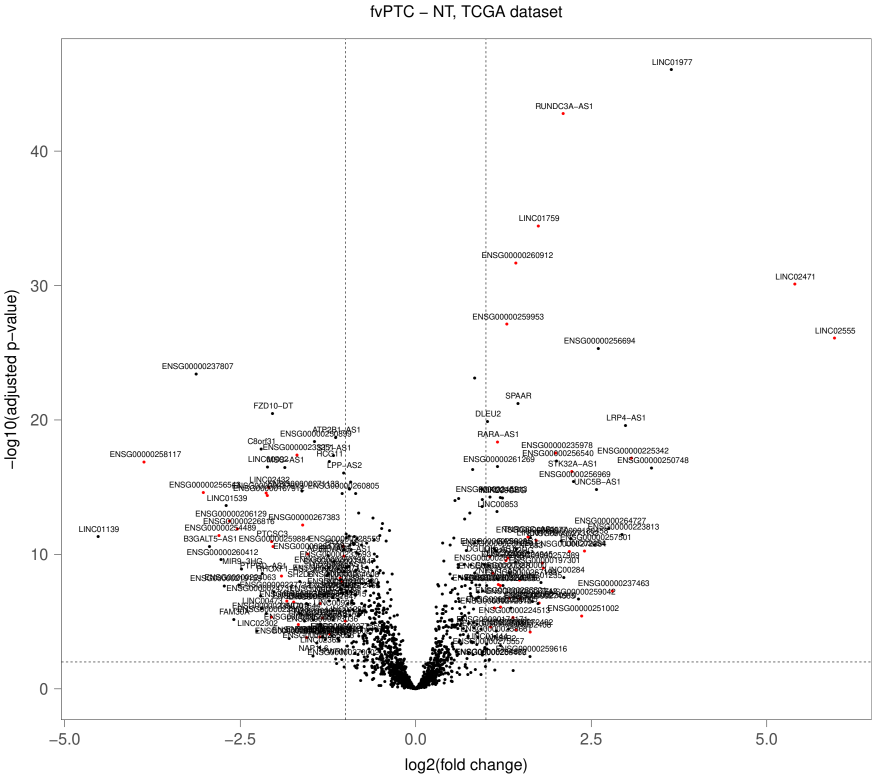

Red highlights lncRNA differently expressed in Microarray, RNASeq PRJEB11591 and TCGA datasets

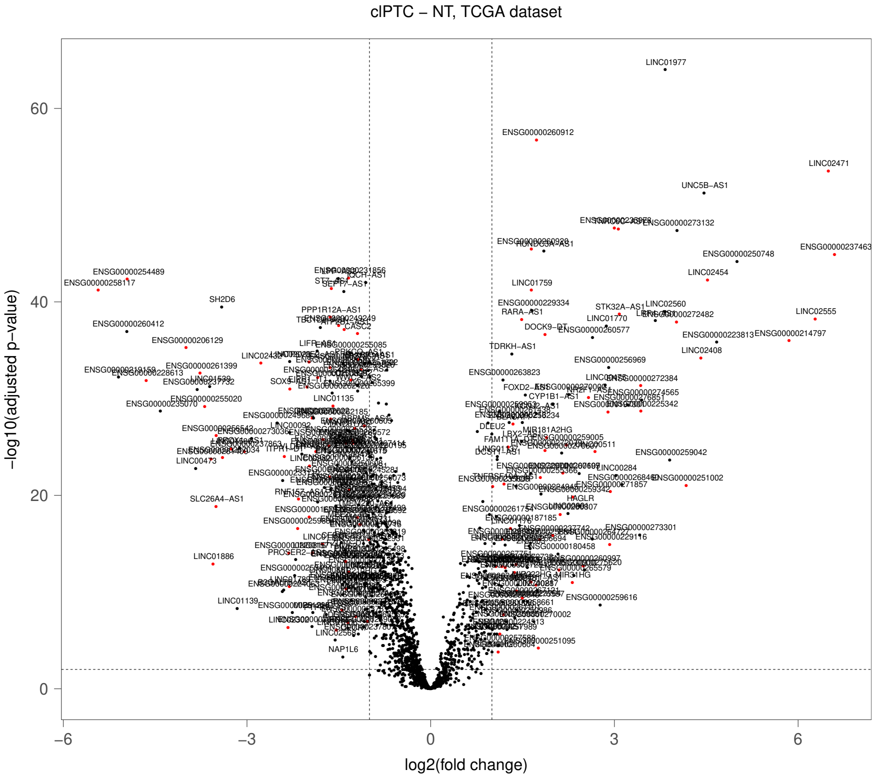

Red highlights lncRNA differently expressed in Microarray, RNASeq PRJEB11591 and TCGA datasets
